# Twelve-Month-Olds' Understanding of Intention Transfer through Communication

**DOI:** 10.1371/journal.pone.0046168

**Published:** 2012-09-24

**Authors:** Him Cheung, Wen Xiao, Ching Man Lai

**Affiliations:** Department of Psychology, The Chinese University of Hong Kong, Shatin, Hong Kong, China; Goldsmiths, University of London, United Kingdom

## Abstract

Do infants understand that intention can be transferred through communication? We answered this question by examining 12-month-olds' looking times in a violation-of-expectation paradigm with two human agents. In familiarization, the non-acting agent spoke, clapped her hands, read aloud a book, or remained silent before the acting agent grasped one (the target) of two objects. During test only the non-actor remained, grasping either the target or distractor. The infants looked longer in the distractor than target condition, suggesting violation of expectation, only if the non-actor had spoken or clapped in familiarization. Because the non-actor never had grasped any of the objects in familiarization, the infants' expectation on her behavior could have developed from the understanding that her intention was transferred to the actor, who executed it by grasping the target in familiarization, via speaking and clapping as acts of communication (but not reading aloud and remaining silent).

## Introduction

Woodward [1] demonstrated that at 6 months infants start to interpret human agents' actions as goal-directed. The experimental paradigm involved an agent consistently grasping one of two objects sitting side by side on a stage in several habituation trials before the objects swapped locations in the test trials. The agent then grasped either the old or new object. The infants looked longer at the apparatus in the new- than old-object condition, which was interpreted as surprise because grasping the new object contradicted the agent's intention or goal suggested by consistent grasping throughout habituation. Further research has shown that infants' understanding of the relationship between the agent and the target object has to be mediated by the more mentalistic concept of personalized goal rather than sheer physical association, because the effect depends on whether the action appears to be intentional or accidental [2], agent identity [3], the agent's prior pursuit of the target [4], and the broader context in which the action occurs [5].

Hence it is clear that from as young as 6 months infants start to make mentalistic interpretations of others' actions, seeing them as goal-directed. In such an attempt they consider the perceptual and epistemological state of the agent as well, which they probably have learned through self-experience [6]. Luo and Baillargeon [7], and Luo and Johnson [8] demonstrated that 12.5- and 6-month-olds, respectively, would regard an agent's consistent reaching for a target object as indicating a preference for it over an alternative only if both objects were visible to the agent during habituation. Further research has shown that from around 12 months on, infants understand the relationship between seeing and knowing, and would expect an agent to behave in a way that is consistent with his or her perceptual and knowledge state [9]–[10]. Imperfect perception under some circumstances would produce a false mental representation of reality, or false belief, on the agent's part, and infants at this age are able to predict the agent's subsequent behavior [11]–[12] and themselves act accordingly on the basis of the agent's false belief [13]. Note that this is accomplished notwithstanding the infant's own accurate representation of reality which is in conflict with the agent's false belief. It is now generally agreed that such developing mentalism emerging at around 6 months is truly representational [14], and that it is developmentally linked to the “theory of mind” (ToM) capacity measured by more verbal means at age 3 or 4 [15]–[17].

Infants' understanding of intention, perception, and knowledge state promotes their social life, and this is most clearly seen in the development of communication behavior. Early sensitivity to the communicative environment is observable at 4 months when infants first show some special interest in their own names being called [18], followed by sensitivity to adult eye gaze [19], and pointing [20]. Infants' responses to these ostensive signals, for which a neural basis has recently been found [21], indicate an understanding and interest in others' focus of attention and the communication that may follow [22]–[26].

Beyond mere orientation to these signals at a behavioral level, some researchers believe that young infants do interpret them in relation to the pragmatic context and link them to the communicator's goal and intention [20], [24]. For instance, Senju and Csibra [27] demonstrated that 6-month-olds would follow an adult's eye gaze as a referential signal only if it was preceded by direct eye contact between the adult and the infant, and infant directed speech. Hence the infant could decide whether an eye gaze bears a communicative intent by looking for cues in the pragmatic context. Southgate, Chevallier, and Csibra [28] showed that 17-month-olds were able to assess from the pragmatic context whether an agent had accurate information about the location of a target object, and interpret accordingly what the agent was referring to in a subsequent communicative act. Grafenhain, Behne, Carpenter, & Tomasello [29] demonstrated that 14-month-olds could follow an experimenter's pointing to a certain location and retrieved a hidden object even when pointing was part of the context, that is, when pointing was directed not to the infant but to a third person, and the infant was only “overhearing” it. Moll, Carpenter, and Tomasello [30] showed that 14-month-olds could assess an experimenter's experience with some objects after seeing the experimenter's interaction with a third person involving the objects, given that they themselves had been involved in similar interaction.

The above discussion points to an early ability to consider others' mental states in infants' understanding of intention behind action and communication. In the second year after birth infants can even interpret communicative acts directed not to themselves in a mentalistic fashion [29], [30]. Given their understanding that there is always intention behind a communicative act, are they also aware that intention itself can be shared among individuals via communication? Buresh and Woodward [3] showed that 13-month-olds did not spontaneously assume shared intention across agents when there was no sign of communication between them. The authors observed in their first study a typical increase in looking time in the new-goal test condition only when object grasping at habituation and test was performed by the same agent, not by different agents. This stood in contrast with the infants' apparent generalization of a linguistic label in their second study. Hence at 13 months infants understand that goals are personalized whereas the semantics of language is shared.

The problem of whether infants are aware that information about intention could be transferred through communication has recently been attempted by Martin, Onishi, and Vouloumanos [31]. In their experiment 12-month-olds watched an agent (the communicator) consistently reach for a target located next to a distractor in familiarization before access to both objects was later denied for her at test. She then uttered a nonsense word to another agent (the recipient) who was free to grasp the objects, and the infants looked longer when the recipient reached for the distractor than the target. Therefore the infants appeared to anticipate the recipient's grasping of the target instead of the distractor presumably because such intention had been transferred through speech from the communicator. Because this result was not obtained with non-speech coughing nor emotional vocalization, the authors argued that at 12 months infants understand the unique information-transferring function of speech.

Using a different procedure, we further investigate infants' understanding of shared intention through communication in the present study. In addition to the primary question of whether 12-month-olds are aware of the communicative function of speech, we further examine what else may constitute communication about one's intention from infants' perspective. Martin et al.'s [31] results showed that 12-month-olds rejected coughing and exclamation as signals carrying information about the communicator's mind, but it was not clear on what basis the infants made such a decision. It was possible that the infants knew why people coughed and exclaimed, i.e., they already attributed these acts to other causes, and thus did not interpret them as communication signals. It was also possible that they knew nothing about coughing and exclamation, nevertheless rejecting them as acts of communication only because they did not sound like speech. Therefore, the question becomes whether 12-month-olds only accept speech as communicative, or they actively look for reasons behind behavior within the total context and evaluate how likely such behavior is meant to be communicative about one's mind. We attempt this problem by using a modified version of the violation-of-expectation paradigm with two human agents and two distinctive objects in the apparatus. In the classic violation-of-expectation paradigm intention is suggested by an agent's consistent grasping of a target object during familiarization. In the present study the grasping action of one agent (the actor) immediately and consistently follows a brief utterance, clapping of hands, or reading aloud from *another* agent (the non-actor) in familiarization. If the infants attribute the actor's grasping to the non-actor's intention which could have been conveyed to the actor through speaking, clapping, or reading aloud, longer looking times would be expected for the distractor than target at test, when only the non-actor remains, grasping either the target or distractor.

We hypothesize that such a pattern of looking time difference would emerge in the speaking condition, consistent with Martin et al.'s [31] findings. Speaking is compared with clapping, which indicates communicative intent [25] but generally does not carry semantic information. Unlike coughing and emotional vocalization which are readily attributable to known causes, clapping is voluntary, has no apparent cause, and thus may appear ambiguous to the infants. But given its social nature [25] and that in the present procedure it is tightly followed by the actor's grasping of the target, it is possible that the infants may interpret it as communication causing the actor to “act out” the non-actor's mind. In other words, the inherent social nature of clapping, its temporal proximity with the actor's subsequent grasping, and its lack of an alternative attribution in the present procedure may suggest to the infants that it could be communicative about the non-actor's mind, causing the actor's subsequent grasping. Reading aloud provides an interesting contrast: It is speech, yet attributable to an apparent external cause, that is, the book. The infants therefore may not view reading as conveying the reader's mind content. Comparing clapping and reading thus enables us to evaluate the importance of being speech (reading) versus not having an apparent non-communicative attribution (clapping) in infants' interpretation of communication signals, when these signals are closely followed by another individual's overt behavior (grasping). Finally, a silence condition is included for comparison, in which the non-actor does not do anything prior to the actor's grasping of the target in familiarization.

## Methods

### Ethics statement

This research was approved by the Ethics Committee, the Social Science Panel, the Chinese University of Hong Kong. The written consent form for parents or caregivers used in this study was also approved by the Ethics Committee.

### Participants

A total of 117 full-term 12-month-old infants were recruited through advertising on a local Internet parent-child forum and subsequently tested. The data from 47 infants were discarded because of one or a combination of the following reasons: fussiness (14); crying (16); experimenter error (1); observer error (1); inter-observer reliability lower than 0.8 (15). Data from the crying and fussy infants were discarded only because their crying and fussiness prevented them from completing the task. Hence the data so discarded were all incomplete data. Decisions to terminate testing due to crying and fussiness were made on the spot by two independent observers, who only saw the infant's face on a television screen in a separate room and were blind to the experimental condition. After data exclusion, the data from the remaining 70 infants were used in the speaking (18; 8 females), clapping (18; 9 females), reading (16; 6 females), and silence condition (18; 8 females) They were all within the age range of 12 months plus or minus 2 weeks, with a mean age of 11.9 months (*SD* = 0.4 months). No caregivers had reported any perceptual, psychological, emotional, or linguistic abnormalities on the part of the infants. All the participants were of Chinese ethnicity, raised in Cantonese-speaking families (i.e., both parents being native Cantonese speakers). Parents' or caregivers' written informed consent on behalf of the infants was obtained before testing. Each participating party was offered approximately USD$6.4 as reimbursement for their travel expenses.

### Design

The present study adopted a 4 (Communication) X 2 (Test) mixed design, with Communication and Test being the between- and within-subject factors, respectively. The infants were randomly assigned to one of the four Communication conditions differing in how two female agents, the non-actor and actor, interacted prior to the actor's grasping of the target object in familiarization. The non-actor spoke to the actor and clapped her hands in the speaking and clapping condition, respectively. In the reading condition, the non-actor held a book and read it aloud, and in the silence condition the two agents did not do anything prior to the actor's grasping of the target. There were two Test conditions (within-subject): old goal versus new goal. In the old goal condition, the non-actor grasped the same target object in the test trial as the actor had done in familiarization. In the new goal condition she grasped the alternative object, or the distractor, in the test trial. Only the non-actor appeared in the test trials.

The present familiarization procedure was modeled after those used by Luo and Baillargeon [7], Luo and Johnson [8], Surian, Caldi, and Sperber [10], and Onishi and Baillargeon [11], which departed from the habituation method used by Woodward [1], in that the infant was familiarized with a certain display with a fixed number of trials instead of being habituated to the display until a criterion for reduced looking was reached. An inter-trial between familiarization and test showing only the alternative objects with swapped locations was also not included. We adopted the familiarization instead of habituation procedure because we were interested in the infants' feeling of surprise when what was displayed at test contradicted what had been established in familiarization, not their recovery of responding from habituation when changes were noticed. The familiarization procedure also ensures equal treatment for individual infants (each receiving a fixed number of trials) and is overall more time efficient. With the habituation method infants could become so inattentive toward the end of a lengthy habituation session that they are no longer engaged with the apparatus for further test. The familiarization procedure is an effective alternative in which infants can encode all the necessary information for evaluating the test events, given its successful use in many previous studies [e.g., 7, 8, 10,11].

### Procedure

The infants received three familiarization and two test trials. Each familiarization trial consisted of a 15-s pre-trial followed by the main procedure; each test trial consisted of a 2-s pre-trial followed by the main procedure. The infant sat on the parent's or caregiver's lap facing a stage 90 cm away; parents and caregivers were instructed to be silent and not to interact with their infants during test. Infant looking behavior was videotaped by a hidden camera mounted at the front of the infant below the stage, and the image being recorded was simultaneously transferred and projected onto a television screen in a separate room. Two naïve, independent observers monitored the infant's looking behavior through this television screen. The observers, separated by a black curtain, were blind to the experimental conditions and could not see the stage, the objects, nor the agents from the screen. They were instructed to press and hold on to a designated button on a game pad linked to a computer when the infant was attending to the stage, and release it when the infant looked away. Infant looking times were thus recorded by the computer, which also calculated an inter-observer reliability for each infant. The looking times from the primary observer was used if the reliability was at 0.8 or above; otherwise the data from the infant were discarded.

#### Familiarization events

At the start of each familiarization trial, a curtain was drawn and the following setup appeared in front of the infant. Two clearly different toy objects (stuffed lion and pig) were aligned 48 cm apart on a stage, one to the right and the other to the left of two female agents sitting behind the stage facing the infant. The agents had their eyes focused on a neutral mark on the stage midway between the two objects. From the infant's perspective, therefore, both agents were looking at and paying attention to the display. The observers were monitoring the infant's looking at the setup in another room through a television screen, and when cumulative looking reached two seconds, a tone was played on the computer which signaled the beginning of the pre-trial. In the speaking condition, the non-actor uttered some Japanese speech while the actor remained still and silent. Since no parents or caregivers had reported any knowledge of Japanese on the infants' part, the speech was assumed to be unintelligible to the infants. Both the actor and non-actor kept looking at the center mark on the stage while the non-actor spoke. They were not looking at each other. We assumed that if the infants regarded speech as communicative, they would infer that the non-actor was talking to the actor about the display, because both agents were looking at and paying attention to it while the non-actor spoke. Although the two agents were not looking at each other, the fact that they were paying attention to the same display while one of them spoke made it likely from the infants' perspective that they were communicating with each other. In the clapping condition, the non-actor clapped her hands at a rate of about one clap per second while both agents kept looking at the center mark on the stage. The agents did not look at each other and the actor remained still and silent. Similar to the speaking condition, the fact that both agents were looking at the same display while clapping was done made it likely that they were communicating with each other about the display, if clapping was interpreted as communication at all. In the reading condition, the non-actor was holding a book, looking at it, and read aloud the same Japanese script as used in the speaking condition. The actor kept looking at the center mark on the stage and remained still and silent. Because the non-actor was looking at the book when uttering the speech whereas the actor was looking at the display, from the infants' perspective it was less likely that the non-actor was expressing her thought about the display to the actor, because the book, to which she paid her attention, provided an explanation, or “source”, for her speech that was external to her mind. The two agents did not look at each other. In the silence condition, both agents remained still and silent, looking at the center mark. They did not look at each other. The pre-trial lasted for 15 seconds.

Immediately after the pre-trial the main familiarization procedure followed, in which the actor turned to, looked at, and then reached for one of the two objects (i.e., the target) on the stage and paused, keeping her hand on it. The non-actor also (put down the book in the reading condition) turned to and looked at the target object while the actor did so, but did not reach for it. Infants' looking at the setup was timed and the main procedure ended if they (1) looked away for two consecutive seconds after having looked at the setup for at least five cumulative seconds, or (2) looked at the setup for 30 cumulative seconds without looking away for two consecutive seconds. The end of the main procedure was signaled by the closing of the curtain in front of the stage. The main procedures in the four Communication conditions were identical.

Cumulative looking times during the pre-trial and main procedure were computed separately. One of the agents always wore a baseball cap so that the infants could more easily differentiate between them. The two agents had no eye contact or any other forms of interaction with each other throughout the experiment. A metronome was used to produce soft beats at one beat per second to help the agents time their actions more accurately.

#### Test events

Two test trials were administered after the three identical familiarization trials. Prior to the first test trial, the two toy objects swapped locations so that the infant's expectation of the non-actor's grasping could not be explained by a simple association with the direction of grasping. In both test trials, only the non-actor remained in the apparatus sitting at the centre position behind the stage. During the 2-s pre-trial, the non-actor turned to, looked at, and reached for either the target as the actor had done in the main familiarization procedure (old goal condition), or the distractor which had not been touched by the actor (new goal condition). In the main procedure that followed, the non-actor kept her hand on the object and eyes focused on it. The main procedure ended if the infant (1) looked away for 2 consecutive seconds after having looked at the setup for at least 5 cumulative seconds, or (2) looked at the setup for 60 cumulative seconds without looking away for 2 consecutive seconds. The other test trial was identical to the first except that the distractor instead of the target was grasped. Cumulative looking times during the pre-trial and main procedure were computed separately. Target versus distractor location, object identity, and old versus new goal test trial order were counterbalanced across the infants in each Communication condition.

## Results

### Reliability

An inter-observer reliability was calculated by the procedure-controlling software for each infant during the main familiarization and test trials, indicating how much the two observers agreed on whether the infant was attending to the stage. The program divided each second into 100-ms intervals and tallied those on which the two observers agreed. Reliability was calculated as the quotient obtained through dividing the number of agreed intervals by the total number of intervals. Overall reliability for the 70 infants was 0.92. This method of reliability calculation, together with the procedure-controlling software, was adopted from Baillargeon, Luo, and their colleagues, which had been used in many of their previous studies [e.g., 7, 8].

### Familiarization

We found no significant looking time differences due to infant gender, object identity, target location, and order of test trials; hence these variables were collapsed for all the subsequent analyses.

Collapsing the four Communication conditions, the average looking times for the first, second, and third familiarization trials were 21.8 s (*SD* = 8.7 s), 19.7 s (*SD* = 8.3 s), and 17.7 s (*SD* = 9.4 s). A repeated-measures analysis of variance (ANOVA) showed that the overall looking time decrement from the first to the third familiarization trial was significant, *F*(2, 138)  = 6.8, *p* = .001, suggesting that the infants were encoding the information presented in the familiarization trials and were getting used (habituated) to it through repetition.

The average looking times in the main familiarization procedure were similar across the four Communication conditions (speaking: *M* = 18.8 s, *SD* = 6.6 s; clapping: *M* = 20.4 s, *SD* = 5.9 s; reading: *M* = 21.1 s, *SD* = 8.2 s; silence: *M* = 18.6 s, *SD* = 7.2 s; *F*(3, 66)  = 0.6, ns.). On the last familiarization trial, the infants looked at the setup for an average of 16.3 s (*SD* = 2.1 s), 19.2 s (*SD* = 2.1 s), 20.1 s (*SD* = 2.5 s), and 15.2 s (*SD* = 2.3 s) in the speaking, clapping, reading, and silence condition, respectively, *F*(3, 66)  = 1.1, ns. These results suggested similar levels of infant attention across the four Communication conditions throughout familiarization.

### Test trials

**Figure 1:**
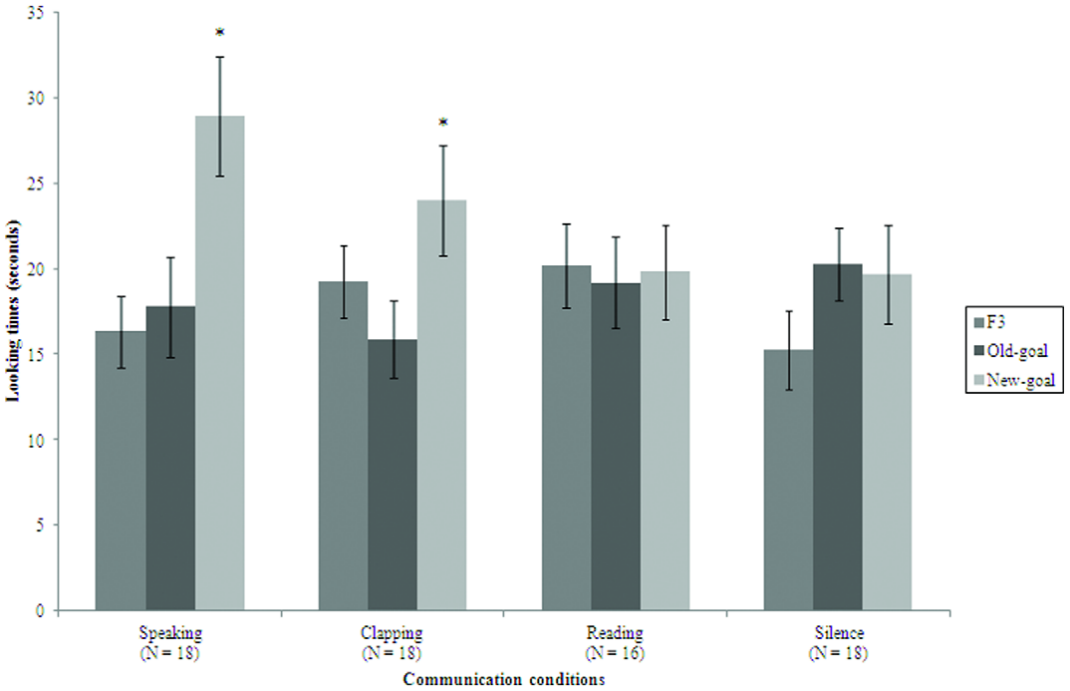
Mean looking times. An asterisk denotes a statistically significant difference between the test trials. Error bars indicate standard errors; F3 is the last familiarization trial.

Looking times in the main test procedure were submitted to a repeated-measures 4 (Communication) X 2 (Test) ANOVA. Mean looking times for the familiarization and test trials in the various conditions are presented in Figure 1. The Test main effect was significant, *F*(1, 66)  = 7.8, *p* = .007; overall mean looking time in the new-goal condition (*M* = 23.1 s, *SD* = 13.4 s) was longer than that in the old-goal condition (*M* = 18.3 s, *SD* = 10.4 s). This main effect was however qualified by the Communication X Test interaction, *F*(3, 66)  = 2.8, *p* = .04. Planned comparisons indicated that the Test simple effect was significant in the speaking (new goal: *M* = 29.0 s, *SD* = 14.8 s; old goal: *M* = 17.8 s, *SD* = 12.3 s; *t*(17)  = 2.6, *p* = .019) and clapping condition (new goal: *M* = 24.0 s, *SD* = 13.8 s; old goal: *M* = 15.9 s, *SD* = 9.7 s; *t*(17)  = 2.5, *p* = .025), but not in the reading (new goal: *M* = 19.8 s, *SD* = 11.1 s; old goal: *M* = 19.2 s, *SD* = 10.7 s; *t*(15)  = 0.3, ns.) and silence condition (new goal: *M* = 19.6 s, *SD* = 12.2 s; old goal: *M* = 20.2 s, *SD* = 9.0; *t*(17)  = 0.18, ns.).

## Discussion

Using behavioral measures that capitalize on infants' increased attention toward expectation-violating events, previous studies have established that infants begin to interpret others' behavior in a mentalistic fashion well before the end of their first year [8], [1]. More advanced belief thinking is evident at around 1.5 years [11]. Communicative behavior is interpreted by young infants as mentalistic too [27], [29]. The present study further demonstrates that 12-month-olds are capable of understanding the very essence of communication, that is, the transmission of ideas and intention. Different forms of possible communicative behavior were investigated: speech in an unfamiliar language which was apparently communicative albeit totally unintelligible; clapping, which was social in nature and could be understood by the infants as carrying information about the non-actor's mind because it did not have an apparent attribution and was closely followed by the actor's grasping of the target; reading aloud, which was speech itself but had an apparent attribution that was external to the mind of the non-actor, that is, the book. These experimental conditions were compared to a silence condition in which there was a lack of activity for both agents prior to the actor's grasping of the target. Results showed that the infants expected the non-actor to grasp the target at test only in the speaking and clapping condition. Therefore, rather than regarding only speech as communicative in a simple and straightforward fashion, 12-month-olds consider the total context in their interpretation of communication signals, including the temporal relationship between a could-be communicative act and another person's subsequent behavior and whether the could-be communicative act is readily explainable by a cause external to the communicator's intention. When such a cause is available, even speech could be considered as not conveying one's mind content (reading); when such a cause is unavailable, even a social act generally not carrying semantic information could be viewed as communicative (clapping).

Together with Martin et al. [31], the present study shows that in a violation-of-expectation paradigm an expectation on an agent's future behavior could develop without him or her performing that behavior in familiarization. The behavior could be performed by another individual yet attributed to the critical agent if there is communication between them. Hence in addition to assigning intention to behavior as already demonstrated in the literature, 12-month-olds also appreciate the lack of a one-to-one relationship between intention and behavior in some situations. Whereas behavior usually implies intention, intention is not always accompanied by a corresponding behavior. Rather, it is transferable through communication and could subsequently be expressed elsewhere behaviorally by another individual.

Our procedure departs from Martin et al.'s [31] in that we had the two agents look at the display while talking instead of looking at each other. Also, the actor did not respond to the non-actor's speaking and clapping in an apparent way except that she reached for the target immediately after these acts. Such discrepancies are due to the different procedures used in the two studies. First, Martin et al.'s [31] design highlighted *who* were involved in the communication (the parties involved looked at each other) whereas the present study emphasized *what* was being talked about (the agents looked at the display while communicating). In our design it was important to highlight the fact that the agents were talking about the two objects on the table, not anything else, because communication happened *before* grasping, that is, before a person-object link was established. With this procedure it might not be immediately obvious to the infants that communication was about the objects if the agents did not look at the display while talking. In contrast, communication happened *after* consistent grasping of the target in Martin et al.'s [31] procedure and thus it was easier for the infants to assume that communication was about the objects even if the agents did not look at them while talking.

Second, more importantly, with the current procedure what was being tested was precisely whether the infants would interpret the actor's grasping as a result of or response to the non-actor's could-be communicative acts, which immediately preceded grasping. Hence, eye contact between the agents and responsive acts on the actor's part such as nodding or the verbal “OK” could not be included in the speaking and clapping conditions because that would have introduced a confound into the design, cueing the infants to interpret speaking and clapping as communication. Because the purpose of the present study is to compare speaking, clapping, and reading aloud *themselves* on their relative likelihoods of being seen by infants as conveying an intention from one mind to another, eye contact between the agents and the actor's responsive acts are considered extraneous cues for communication that confound the results. Note that we do not reject eye contact and recipient responsive acts as ordinary communication cues for infants; we do not include them in this study only because we are more interested in the acts of speaking, clapping, and reading aloud themselves devoid of such cues.

We consider some alternative ways of thinking about the present results. First, speaking and clapping in combination with looking at the display may suggest to the infants that the non-actor is somehow particularly aware of or interested in what the actor is doing and therefore likely to copy it in the test trials. Nevertheless, the procedural fact that speaking and clapping happen *before* the actor's grasping makes this interpretation not very plausible. Because of the temporal arrangement of the events, speaking and clapping are more likely to be the cause rather than result of the actor's grasping. Second, speaking and clapping may indicate the non-actor's particular interest in the actor (the person, not her action), so that copying of her behavior becomes more likely in the test trials. We think that this possibility is greatly reduced by that fact that the non-actor looks at the display rather than the actor in familiarization. Third, the infants may regard people who communicate with one another as more likely to have common goals. Under this interpretation, the infants do interpret speaking and clapping, not reading aloud, as communication, but what is being communicated is not necessarily the non-actor's intention and has nothing to do with the actor's grasping. Again, we think that the temporal proximity between these communicative acts and the actor's subsequent grasping makes it apparent that the communication has something to do with the grasping.

In sum, the current study complements Moll et al. [30], Grafenhain et al. [29], and Martin et al. [31] by showing that infants as young as 12 months old are sensitive to others' communicative acts and understand the information-transferring nature of such acts. Infants do not only regard speech as communicative in a mechanical way; they look for cues in the total context to define communication. Thus young infants interpret their communicative environment proactively in the constant process of making sense of the social world. We have yet to explore what aspects of the infants' own social experience may have contributed to such early understanding [6], and perhaps what early knowledge about the physical world may have laid the foundation for it.
